# Elevated interferon-stimulated gene transcription in peripheral blood mononuclear cells occurs in patients infected with genotype 1 but not genotype 3 hepatitis C virus

**DOI:** 10.1111/jvh.12310

**Published:** 2014-09-09

**Authors:** M W Robinson, R Swann, A Sigruener, S T Barclay, P R Mills, J McLauchlan, A H Patel

**Affiliations:** 1 MRC – University of Glasgow Centre for Virus ResearchGlasgow, UK; 2 Gartnavel General Hospital, NHS Greater Glasgow and ClydeGlasgow, UK; 3 Institute of Clinical Chemistry and Laboratory Medicine, Regensburg University Medical CenterRegensburg, Germany; 4 Glasgow Royal Infirmary, NHS Greater Glasgow and ClydeGlasgow, UK; † School of Biochemistry and Immunology, Trinity College DublinDublin, Ireland

**Keywords:** genotype, HCV, IFN, interferon-stimulated genes, transcriptomics

## Abstract

Hepatitis C virus (HCV) can be classified into seven distinct genotypes that are associated with differing pathologies and respond differently to antiviral therapy. In the UK, genotype 1 and 3 are present in approximately equal proportions. Chronic infection with HCV genotype 3 is associated with increased liver steatosis and reduced peripheral total cholesterol levels, which potentially influences peripheral immune responses. To understand these differences, we investigated host gene transcription in peripheral blood mononuclear cells by microarray and quantitative PCR in patients with genotype 1 (*n *=* *22) or genotype 3 infection (*n *=* *22) and matched healthy controls (*n *=* *15). Enrichment of genes involved in immune response and inflammatory pathways were present in patients infected with HCV genotype 1; however, no differences in genes involved in lipid or cholesterol metabolism were detected. This genotype-specific induction of genes is unrelated to *IL28B* genotype or previous treatment failure. Our data support the hypothesis that genotype 1 infection drives a skewed Type I interferon response and provides a foundation for future investigations into the host–pathogen interactions that underlie the genotype-specific clinical outcomes of chronic HCV infection.

## Introduction

Hepatitis C virus (HCV) is a highly diverse human pathogen that is prevalent worldwide. The virus has been classified into seven distinct genotypes, which are approximately 30% divergent at the nucleotide sequence level [1]. The genotypes segregate according to geographical distribution, with certain genotypes widely distributed across the globe. In the UK, genotypes 1 and 3 (gt1 and gt3, respectively) predominate and have approximately equivalent prevalence [2,3]. From a clinical point of view, the distribution of HCV genotypes is of great importance due to the distinctive clinical outcomes associated with gt1 or gt3 infection.

The response to antiviral therapies is influenced by HCV genotype. Gt1 infections have a lower sustained virological response (SVR) rate when treated with combination pegylated interferon (IFN)*α* and ribavirin (40–50% compared to 70–80% for gt3) [4,5]. This effect of HCV genotype on treatment outcome differs depending on the specific antiviral drugs employed. A recent phase III clinical trial of the pan-genotypic direct acting antiviral sofosbuvir identified a higher rate of relapse in gt3-infected patients, suggesting that gt3 HCV may be relatively more difficult to treat when using IFN-free regimes [6]. HCV also exerts genotype-specific effects on host cell lipid metabolism. Gt3 infections are associated with an increased prevalence of liver steatosis and reduced serum cholesterol concentration, both of which resolve following successful viral clearance [7–10]. These genotype-specific clinical manifestations are independent of known negative predictors of treatment response [5] and co-morbidities associated with liver steatosis [10].

The genotype-specific decrease in peripheral serum cholesterol levels provides a potential mechanism through which HCV could modulate peripheral immune cells in a genotype-specific manner during chronic infection. Peripheral cholesterol levels are known to play an important role in regulating proinflammatory immune responses within immune cells [11,12]. This raises the possibility that gt3 HCV, by reducing peripheral cholesterol levels, also reduces the proinflammatory effects exerted by peripheral immune cells. Studies investigating the interaction between chronic HCV infection and peripheral blood immune cells have identified a proinflammatory signature associated with elevated interferon-stimulated genes (ISGs) [13], as well as elevated expression of proinflammatory cytokines [14]. However, these studies have focussed exclusively on infection with gt1 HCV and fail to address potential genotype-specific peripheral blood mononuclear cell (PBMC) responses. In this study, we set out to explore distinctive transcriptional profiles induced by different HCV genotypes in PBMCs.

## Materials and Methods

### Clinical samples

Clinical samples were used with informed consent conforming to the ethical guidelines of the 1975 Declaration of Helsinki and study protocols were approved by the West of Scotland Research Ethics Committee. Blood samples were obtained from a cohort of patients with chronic hepatitis C and healthy volunteers (Table1). Exclusion criteria included co-infection with other viruses (hepatitis B virus or HIV), non-HCV related liver disease, active antiviral therapy, metabolic disorders, medication affecting lipid metabolism, current intravenous drug use, chronic alcohol use and a body mass index (BMI) >30. Participants were assessed for HCV risk factors, HCV infection status (anti-HCV antibodies, viral load and virus genotype) and markers of hepatic inflammation/cirrhosis (alanine aminotransferase levels and transient elastography [FibroScan]). Participants were classified as having no/minimal fibrosis (FibroScan score <7.1 kPa), fibrosis (FibroScan score 7.1–12.5 kPa) or cirrhosis (FibroScan score >12.5 kPa) [15]. Statistical significance was calculated using GraphPad Prism 5 (GraphPad Software, La Jolla, CA, USA).

**Table 1:** Clinical data

	Healthy controls	Genotype 1	Genotype 3
*n*	15	22	22
Male:Female	10:5	15:7	19:3
Caucasian:Asian	15:0	22:0	19:3
Age	44.0 (37.1–50.9)	45.1 (42.5–47.7)	40.6 (37.7–43.6)
BMI*	25.2 (23.2–27.1)	25.4 (24.3–26.6)	25.1 (23.5–26.7)
rs12979860 (CC:CT:TT)	n/d	5:14:3	6:8:2†
Previous Treatment Failure (Y:N)	n/a	11:11	4:18
Viral Load (1 × 10^6^ IU/mL)*	n/a	1.5 (0.7–2.2)	2.1 (0.6–3.6)
FibroScan (kPA)	n/d	13.7 (6.4–21.1)	14.6 (7.3–21.9)
Serum ALT (IU/mL)	33.5 (23.6–43.4)	110.6 (41.6–179.6)‡	127.7 (46.0–209.5)‡
Serum Cholesterol (mmol/L)	5.4 (5.0–5.8)	5.0 (4.6–5.4)	3.9 (3.4–4.4)‡§
Serum Total/HDL Cholesterol	4.6 (3.9–5.2)	3.8 (3.3–4.3)	3.9 (3.2–4.6)
Haemoglobin (g/L)	146 (135–157)	150 (143–156)	150 (140–159)
Platelets (1 × 10^9^/L)	265 (216–313)	232 (186–278)	189 (162–217)‡
White Cell Count (1 × 10^9^/L)	6.6 (6.0–7.3)	6.4 (5.6–7.2)	6.7 (5.8–7.5)
Lymphocytes (1 × 10^9^/L)	2.0 (1.8–2.3)	2.2 (1.8–2.6)	2.1 (1.8–2.4)
Neutrophils (1 × 10^9^/L)	3.9 (3.3–4.4)	3.5 (2.9–4.0)	3.9 (3.2–4.5)

All values are given as the mean with the 95% confidence interval in brackets with n/a signifying not applicable and n/d signifying not done.

* For viral load *n *=* *41 (22 gt1 and 21 gt3); for BMI *n *=* *57 (15 healthy control, 21 gt1 and 21 gt3).

† Genotype information was unavailable for six gt3-infected patients. Significant differences (*P*-value<0.05) are denoted as

‡ versus the ‘Healthy Control’ group or

§ versus the ‘Genotype 1’ group.

### RNA extraction

RNA was extracted from PBMCs following separation over a density gradient (Histopaque®-1077; Sigma-Aldrich, Gillingham, UK). Total RNA was extracted using RNeasy Minicolumns with on-column DNase-digestion (Qiagen, Manchester, UK) following manufacturer's guidelines. RNA quality and quantity were assessed by NanoDrop 1000 (Thermo Scientific) (Thermo Fisher Scientific, Waltham, MA, USA) and 2200 TapeStation (Agilent Technologies, Santa Clara, CA, USA) instruments.

### Microarray analysis

RNA (150 ng) was hybridized onto modified Agilent 4 × 44K microarrays containing 201 additional probes, corresponding to 119 genes previously not found on the array (Agilent Technologies, Santa Clara, CA, USA). Raw data were extracted using Feature Extraction software (Agilent Technologies, Santa Clara, CA, USA), and the generated text files were processed and analysed with ChipInspector (Genomatix Software, Munich, Germany). Differentially regulated genes were selected with a cut-off fold change of 2 (in either direction) and a signal intensity of >100 in one of the biological groups.

### Clustering and gene-annotation enrichment analysis

Gene-annotation enrichment analysis was carried out with DAVID Bioinformatics Resource 6.7 [16], using the Agilent HumanGenomeCGH44K background gene set (for microarray data), and adjusting for multiple testing by the Benjamini–Hochberg procedure. Data were clustered in R using the log-transformed microarray signal values with the hclust package (utilizing agglomerative clustering) and visualized by heatmap.2 (from the gplots package).

### Reverse transcription and quantitative real-time PCR (Q-PCR)

Total RNA was reverse transcribed with the SuperScript® VILO™ cDNA Synthesis kit (Life Technologies, Paisley, UK) according to manufacturer's instructions. Microarray results were validated by Q-PCR using TaqMan® assays (Life Technologies, Paisley, UK; Table S1). *HPRT1* was used as a reference gene, and data were analysed using the comparative threshold cycle method [17]. Statistical significance was calculated using the nonparametric Kruskal–Wallis test with Dunn's multiple comparison post-test using GraphPad Prism 5.

## Results

### Genotype-specific transcriptional changes are observed in known ISGs but not genes related to lipid metabolism

**Figure 1:**
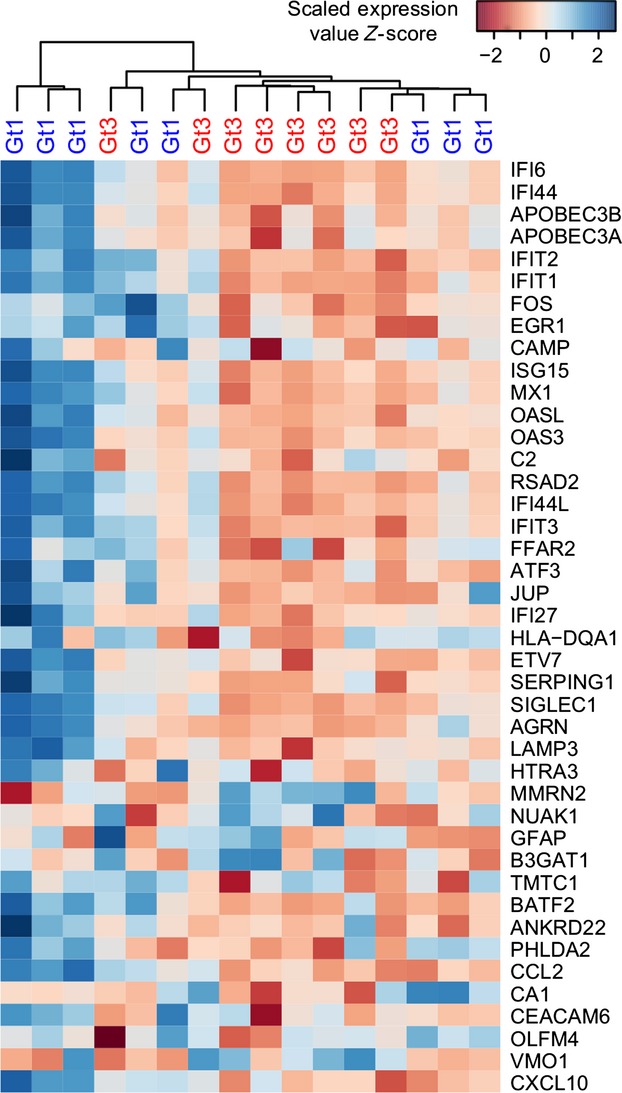
Heat map of genes identified as differentially expressed between gt1- (*n *=* *8; blue labels) and gt3-infected patients (*n *=* *8; red labels). Microarray signal values were log-transformed, scaled using *Z*-score (dark blue denotes higher and dark red denotes lower), and then clustered based on Euclidean distance metrics using the hclust R package.

To investigate potential genotype-specific differences in PBMC transcription, we selected a subgroup of chronically infected patients (gt1 *n *=* *8 and gt3 *n *=* *8) who were matched by age, gender and had low FibroScan scores (to exclude any transcriptional changes associated with fibrosis or cirrhosis). We utilized Agilent 4 × 44K microarrays modified to detect additional genes, involved in lipid metabolic processes that are absent in the original array. A total of 42 differentially expressed genes were identified; 37 were up-regulated in the gt1 samples and 5 were up-regulated in the gt3 samples (Fig.1 and Table S2). This small number of differentially expressed genes highlights the subtle difference between chronic infection with either HCV gt1 or gt3. Sample clustering on the basis of the log-transformed microarray signal values from these 42 differentially regulated genes failed to separate the different genotypes although a subgroup of gt1-infected samples did form a distinct cluster (Fig.1). This cluster was statistically significant when analysed by multiscale bootstrap resampling (data not shown). This implies that at least a proportion of patients infected with gt1 HCV are characterized by a distinct peripheral transcriptional profile.

The majority of the differentially expressed genes are known ISGs and play important roles in pro-inflammatory immune responses to viral infections. The largest differential transcription was observed for interferon alpha-inducible protein 27 (IFI27; up-regulated 6.0-fold in HCV gt1 infection; Table S2). Sarasin-Filipowicz and colleagues previously identified genotype-specific transcription of IFI27 in liver biopsies [18]; however, reports are conflicting on whether IFI27 transcription is also up-regulated in PBMC samples from patients with chronic HCV infection [13,14]. No genes relating to lipid metabolism were identified as differentially expressed between HCV gt1 and gt3 infection, and only one gene related to lipid signalling was identified – free fatty acid receptor 2 (FFAR2; up-regulated 2.2-fold in HCV gt1 infection; Table S2), a G-protein-coupled receptor, expressed on leucocytes, that recognizes short-chain free fatty acids [19].

### Enrichment of immune-related pathways and Type I IFN-responsive genes in gt1-infected patients

**Figure 2:**
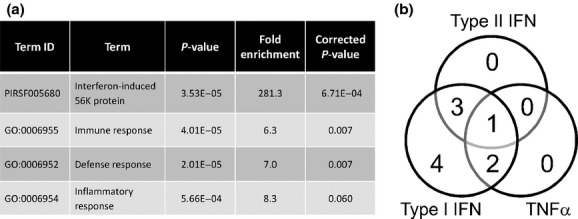
Pathway analysis of the differentially expressed genes identified from microarray analysis (gt1, *n *=* *8; gt3, *n *=* *8). (a) Enriched terms from DAVID analysis ranked by corrected *P*-value. (b) Classification of identified IFN-stimulated genes using published sets of genes induced by Type I IFN, Type II IFN or TNF*α* in PBMC [21].

To gain further functional understanding of these genotype-specific differences, we performed gene-annotation enrichment analysis. Significant enrichment of gene terms relating to antiviral pathways and innate immune responses were identified amongst the 42 differentially transcribed genes (Fig.2a). In particular, the gt1-infected patients demonstrated an enrichment of the immune response pathway, the inflammatory response pathway and the antiviral interferon-induced 56K protein family [20] (Fig.2a). Although decreased peripheral cholesterol levels were observed in the gt3-infected patients (Table1), no enrichment of lipid or cholesterol metabolic pathways was observed.

To further analyse these differentially transcribed genes, we matched the ISGs to published gene sets generated following stimulation of PBMCs with Type I IFN, Type II IFN, interleukin (IL)12 or TNF*α*, respectively [21]. The ISGs identified as having genotype-specific expression were all classified as being induced by Type I IFN, and genes induced exclusively by Type II IFN or TNF*α* were absent (Fig.2b).

### Patients infected with HCV gt1 have elevated peripheral ISG transcription but comparable IFN transcription to gt3-infected patients

To validate the microarray findings, we selected three of the identified genotype-specific genes (interferon-induced protein with tetratricopeptide repeats 1 (*IFIT1*), ISG15 ubiquitin-like modifier (*ISG15*) and radical S-adenosyl methionine domain containing 2 (*RSAD2*)) and analysed their expression by Q-PCR in the full cohort of patients chronically infected with either HCV gt1 (*n *=* *22) or gt3 (*n *=* *22). We also analysed a group of age- and gender-matched uninfected controls (*n *=* *15). The infected patients showed no significant differences in viral load, liver function tests or FibroScan scores. Gt3 infection was associated with significantly reduced serum cholesterol compared to the uninfected controls and gt1 infection, consistent with previous studies [7–10], as well as a significantly reduced platelet count compared to the uninfected controls (Table1).

**Figure 3:**
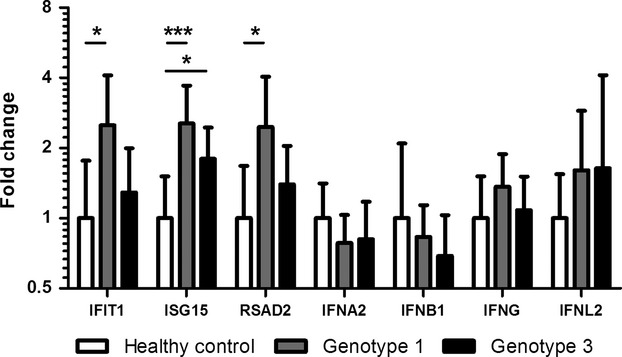
Validation of microarray results and transcription of IFNs using Q-PCR in gt1- (*n *=* *22) and gt3-infected patients (*n *=* *22), and healthy controls (*n *=* *15). For *IFNA2 n *=* *17 gt1, 19 gt3 and 11 healthy controls; for *IFNB1 n *=* *10 gt1, 10 gt3 and six healthy controls; and for *IFNL2 n *=* *8 gt1, 10 gt3 and eight healthy controls. Plots show geometric mean with 95% confidence interval. *denotes *P*-value <0.05 and ***denotes *P*-value <0.001.

We detected a statistically significant increase in transcription of *IFIT1*,*ISG15* and *RSAD2* in gt1-infected patients compared to healthy controls, which was absent or reduced in gt3-infected patients (Fig.3). This elevated transcription of ISGs in gt1-infected patients (geometric mean fold change vs healthy controls: *IFIT1 *=* *2.50, *ISG15 *=* *2.55 and *RSAD2 *=* *2.46) validates the microarray results and demonstrates an association between HCV gt1 and elevated transcription of pro-inflammatory ISGs in the periphery.

To explore the possibility that elevated expression of IFNs in PBMCs induces the ISGs in chronically infected patients, we profiled the transcription of the Type I IFNs *IFNA2* and *IFNB1*, the Type II IFN *IFNG*, and the Type III IFN *IFNL2*. No significant differences were observed in the transcription levels of any of the IFNs between the three groups (Fig.3). The transcription of both Type I and Type III IFNs were very low across all groups, and indeed transcription was undetectable in a number of samples, suggesting that IFNs are unlikely to be driving the elevated ISG transcription in gt1-infected patients.

### Genotype-specific transcriptional profiles are independent of previous treatment failure and unfavourable IL28B genotype

Previous studies have identified elevated ISG expression in the liver [18,22] and PBMCs [23] as predictors of response to IFN outcome. This elevated ISG expression is also associated with the unfavourable *IL28B* ‘CT’ and ‘TT’ genotypes [24]. We therefore sought to examine whether the differences we observed between gt1 and gt3 infections were simply due to differences in the number of individuals with previous treatment failure or in the balance of *IL28B* genotypes.

**Figure 4:**
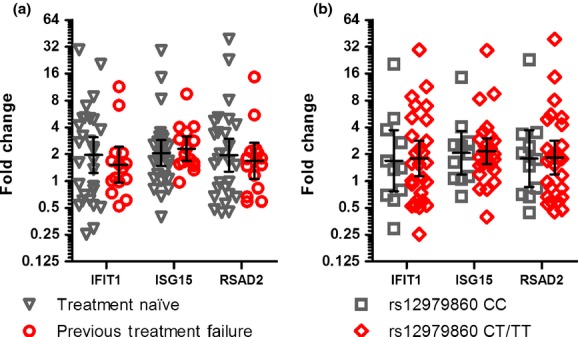
Transcription of genotype-specific genes *IFIT1*,*ISG15* and *RSAD2*. (a) Impact of previous treatment failure comparing treatment naive patients (*n *=* *29) and patients who have previously failed IFN-based therapy (*n *=* *15). (b) Impact of *IL28B* genotype comparing patients with the favourable ‘CC’ genotype (*n *=* *11) and patients with the unfavourable ‘CT/TT’ genotypes (*n *=* *27). Plots show geometric mean with 95% confidence interval.

The number of patients who had previously failed IFN-based therapy was larger in the gt1-infected group compared to the gt3-infected group (11 *vs* 4; *P*-value = 0.055), as was expected based on the genotypic differences in treatment response rates. Despite this, the transcription levels of *IFIT1*,*ISG15* and *RSAD2* between previous treatment failures *vs* treatment naïve individuals were similar (Fig.4a). The gt1- and gt3-infected patient groups had similar proportions of individuals with favourable ‘CC’ and unfavourable ‘CT/TT’ genotypes (Table1). No differences in the transcription levels of *IFIT1*,*ISG15* and *RSAD2* were observed between individuals with favourable or unfavourable *IL28B* genotypes (Fig.4b). These results imply that HCV gt1 may influence peripheral immune cells and induce elevated ISG transcription independent of the described effects of treatment response phenotype or *IL28B* genotype.

## Discussion

Profiling the transcriptional changes in PBMCs associated with specific HCV genotypes illuminates genotype-specific host–pathogen interactions underlying the known clinical features of chronic HCV infection associated with particular genotypes. Using a group of age- and gender-matched patients, selected on the basis of low FibroScan scores to minimize any changes associated with fibrosis or cirrhosis, we identified distinct changes in ISG and immune-related gene transcription within PBMCs from patients infected with either HCV gt1 or gt3. Chronic infection with HCV gt1 was associated with elevated transcription of genes induced by Type I IFNs and involved in immune response and inflammatory response pathways.

Our results confirm previous studies that have identified the presence of ongoing proinflammatory gene induction in PBMCs from patients with chronic HCV infection [13,14]. Furthermore, our results demonstrate that this proinflammatory induction is exaggerated in patients infected with HCV gt1. Importantly, the elevated transcription we observed is unrelated to differences in the proportion of individuals with ‘favourable’ or ‘unfavourable’ *IL28B* genotype or the proportion of patients who have previously failed IFN-based therapy, both of which are associated with elevated expression of ISGs [25].

The ISGs identified in our study with elevated transcriptional in PBMCs from patients with gt1 infection include a number of genes that have previously been identified in transcriptomic studies of HCV-infected liver biopsies [18,22,25,26]. These studies compared liver transcriptomes between patients who achieved SVR with patients who failed to respond to IFN-based therapies and demonstrated that higher expression of ISGs is associated with poor response to therapy [18,22,25,26]. Indeed, pre-existing induction of ISGs predicts treatment response with greater accuracy than the widely used *IL28B* genotype [25]. While these studies failed to specifically address genotype-specific transcriptional changes within the liver, when treatment response was excluded, viral genotype had the next biggest influence on differential gene transcription [22].

In the liver, the elevated transcription of ISGs includes a number of negative regulators of IFN*α* signalling pathways, such as ubiquitin-specific peptidase 18 (*USP18*), which inhibits further IFN signalling and is thought to contribute to the lack of IFN*α*-induced gene induction in treatment nonresponders [27,28]. Strong IFN responses have also been hypothesized to limit the development of robust T-cell immune responses during the establishment of chronic HCV infection [29]. Our results suggest that a general elevation of PBMC ISG transcription occurs in gt1-infected patients. This is in agreement with the conclusion by Sarasin-Filipowicz and colleagues that patients infected with HCV gt1 more frequently have increased expression of ISGs in the liver, which provides a plausible explanation for the relatively poor response to IFN-based therapies in these patients [18]. It is perhaps noteworthy that gt1 infection is associated with spontaneous viral clearance during acute infection [30]. The strong pro-inflammatory responses induced by HCV gt1 may aid clearance during early acute infection but hinder IFN signalling once chronic infection is established.

Despite observing a significant level of hypocholesterolemia in gt3-infected patients, we did not observe changes in genes involved in lipid metabolism. While *in vitro* studies have identified alterations in metabolic gene expression induced by HCV gt3 proteins [31,32], our results demonstrate that such transcriptional changes are not apparent in PBMCs. It is possible that changes in lipid metabolism may only occur within liver hepatocytes despite systemic reduction in total cholesterol levels. Alternatively, genotype-specific differences in lipid metabolism may only be evident at a post-transcriptional level. Clark and colleagues have previously identified a gt3-specific decrease in the serum levels of metabolites involved in the late stages of cholesterol synthesis, which resolve upon successful response to therapy, indicating that HCV gt3 is able to directly perturb cholesterol biosynthesis at a post-transcriptional level [33].

While the elevation of ISG transcription is evident in PBMC samples from gt1-infected patients, the mechanisms underlying this induction are not understood. A key question raised from our study is why gt1 and gt3 mediate different transcriptional responses? Although it is likely that reduced peripheral cholesterol levels act to reduce pro-inflammatory responses of peripheral immune cells, as described previously [11,12], this fails to explain potential genotype-specific differences observed in transcriptomic studies of the liver [18,22]. No differences in the transcription of Type I, II, or III IFNs were observed between any of the study groups, suggesting that alternative immune signalling pathways drive the induction of ISGs. Future studies should address whether gt1- or gt3-specific genetic sequences are responsible for driving elevated ISG transcription.

In summary, our data are consistent with the hypothesis that gt1 infection drives a skewed Type I IFN response. These transcriptional differences may shed light on possible mechanisms underlying the distinctive clinical manifestations of these two HCV genotypes. Our study lays the foundation for further analysis of dysregulation of host cell factors by the distinct HCV genotypes.

## Abbreviations

gt1: genotype 1
gt3: genotype 3
HCV: hepatitis C virus
IFN: interferon
IL: interleukin
ISG(s): IFN-stimulated gene(s)
PBMC(s): peripheral blood mononuclear cell(s)
Q-PCR: quantitative PCR
SVR: sustained virological response

## Supporting Information

**Table S1.**Gene selection for Q-PCR assays. **Table S2.** Genes differentially expressed in gt1-version gt3-infected patients.

## References

[1] Smith DB, Bukh J, Kuiken C (2014). Expanded classification of hepatitis C virus into 7 genotypes and 67 subtypes: Updated criteria and genotype assignment web resource. Hepatology.

[2] Gravitz L (2011). A smouldering public-health crisis. Nature.

[3] Harris KA, Gilham C, Mortimer PP, Teo CG (1999). The most prevalent hepatitis C virus genotypes in England and Wales are 3a and 1a. J Med Virol.

[4] Zeuzem S, Berg T, Moeller B (2009). Expert opinion on the treatment of patients with chronic hepatitis C. J Viral Hepat.

[5] Fried MW, Shiffman ML, Reddy KR (2002). Peginterferon alfa-2a plus ribavirin for chronic hepatitis C virus infection. N Engl J Med.

[6] Lawitz E, Mangia A, Wyles D (2013). Sofosbuvir for previously untreated chronic hepatitis C infection. N Engl J Med.

[7] Bochud P-Y, Cai T, Overbeck K (2009). Genotype 3 is associated with accelerated fibrosis progression in chronic hepatitis C. J Hepatol.

[8] Mihm S, Fayyazi A, Hartmann H, Ramadori G (1997). Analysis of histopathological manifestations of chronic hepatitis C virus infection with respect to virus genotype. Hepatology.

[9] Poynard T, Ratziu V, McHutchison J (2003). Effect of treatment with peginterferon or interferon alfa-2b and ribavirin on steatosis in patients infected with hepatitis C. Hepatology.

[10] Rubbia-Brandt L, Quadri R, Abid K (2000). Hepatocyte steatosis is a cytopathic effect of hepatitis C virus genotype 3. J Hepatol.

[11] Yvan-Charvet L, Welch C, Pagler T (2008). Increased inflammatory gene expression in ABC transporter-deficient macrophages: free cholesterol accumulation, increased signaling via toll-like receptors, and neutrophil infiltration of atherosclerotic lesions. Circulation.

[12] Murphy AJ, Woollard KJ, Hoang A (2008). High-density lipoprotein reduces the human monocyte inflammatory response. Arterioscler Thromb Vasc Biol.

[13] Bolen CR, Robek MD, Brodsky L (2013). The blood transcriptional signature of chronic hepatitis C virus is consistent with an ongoing interferon-mediated antiviral response. J Interferon Cytokine Res.

[14] Kottilil S, Yan MY, Reitano KN (2009). Human immunodeficiency virus and hepatitis C infections induce distinct immunologic imprints in peripheral mononuclear cells. Hepatology.

[15] Boursier J, de Ledinghen V, Zarski J-P (2012). Comparison of eight diagnostic algorithms for liver fibrosis in hepatitis C: new algorithms are more precise and entirely noninvasive. Hepatology.

[16] Huang DW, Sherman BT, Lempicki R (2009). Systematic and integrative analysis of large gene lists using DAVID bioinformatics resources. Nat Protoc.

[17] Schmittgen TD, Livak KJ (2008). Analyzing real-time PCR data by the comparative C(T) method. Nat Protoc.

[18] Sarasin-Filipowicz M, Oakeley EJ, Duong FHT (2008). Interferon signaling and treatment outcome in chronic hepatitis C. Proc Natl Acad Sci USA.

[19] Nilsson NE, Kotarsky K, Owman C, Olde B (2003). Identification of a free fatty acid receptor, FFA2R, expressed on leukocytes and activated by short-chain fatty acids. Biochem Biophys Res Commun.

[20] Fensterl V, Sen GC (2011). The ISG56/IFIT1 gene family. J Interferon Cytokine Res.

[21] Waddell SJ, Popper SJ, Rubins KH (2010). Dissecting interferon-induced transcriptional programs in human peripheral blood cells. PLoS One.

[22] Chen L, Borozan I, Sun J (2010). Cell-type specific gene expression signature in liver underlies response to interferon therapy in chronic hepatitis C infection. Gastroenterology.

[23] Pham TNQ, Lin DMH, Mulrooney-Cousins PM (2013). Hepatitis C virus load and expression of a unique subset of cellular genes in circulating lymphoid cells differentiate non-responders from responders to pegylated interferon alpha-ribavirin treatment. J Med Virol.

[24] McGilvray I, Feld JJ, Chen L (2012). Hepatic cell-type specific gene expression better predicts HCV treatment outcome than IL28B genotype. Gastroenterology.

[25] Dill MT, Duong FHT, Vogt JE (2011). Interferon-induced gene expression is a stronger predictor of treatment response than IL28B genotype in patients with hepatitis C. Gastroenterology.

[26] Asselah T, Bieche I, Narguet S (2008). Liver gene expression signature to predict response to pegylated interferon plus ribavirin combination therapy in patients with chronic hepatitis C. Gut.

[27] Dill MT, Makowska Z, Duong FHT (2012). Interferon-*γ*-stimulated genes, but not USP18, are expressed in livers of patients with acute hepatitis C. Gastroenterology.

[28] Francois-Newton V, Livingstone M, Payelle-Brogard B, Uzé G, Pellegrini S (2012). USP18 establishes the transcriptional and anti-proliferative interferon *α**β* differential. Biochem J.

[29] Park S-H, Rehermann B (2014). Immune responses to HCV and other hepatitis viruses. Immunity.

[30] Grebely J, Page K, Sacks-Davis R (2013). The effects of female sex, viral genotype, and IL28B genotype on spontaneous clearance of acute hepatitis C Virus infection. Hepatology.

[31] Blackham S, Baillie A, Al-Hababi F (2010). Gene expression profiling indicates the roles of host oxidative stress, apoptosis, lipid metabolism, and intracellular transport genes in the replication of hepatitis C virus. J Virol.

[32] Woodhouse SD, Narayan R, Latham S (2010). Transcriptome sequencing, microarray, and proteomic analyses reveal cellular and metabolic impact of hepatitis C virus infection in vitro. Hepatology.

[33] Clark PJ, Thompson AJ, Vock DM (2012). Hepatitis C virus selectively perturbs the distal cholesterol synthesis pathway in a genotype-specific manner. Hepatology.

