# Supplementary material for: Elevated interferon-stimulated gene transcription in peripheral blood mononuclear cells occurs in patients infected with genotype 1 but not genotype 3 hepatitis C virus
Source: J Viral Hepat. 2014 Sep 9;22(4):384–90. doi: 10.1111/jvh.12310 (PMC4409080; doi:10.1111/jvh.12310)
Supplement: Supplementary file 1 — Table S1.Gene selection for Q-PCR assays. Table S2. Genes differentially expressed in gt1-version gt3-infected patients. [file jvh0022-0384-sd1.docx]

**Supplementary Table 1: Gene selection for Q-PCR assays**

| HGNC Gene Symbol | Gene Name | Applied Biosystems TaqMan Assay |
| --- | --- | --- |
| *HRPT1* | Hypoxanthine phosphoribosyltransferase 1 | 4333768F |
| *IFIT1* | Interferon-induced protein with tetratricopeptide repeats 1 | Hs01911452_s1 |
| *ISG15* | ISG15 ubiquitin-like modifier | Hs01921425_s1 |
| *RSAD2* | Radical S-adenosyl methionine domain containing 2 | Hs00369813_m1 |
| *IFNA2* | Interferon, alpha 2 | Hs00265051_s1 |
| *IFNB1* | Interferon, beta 1 | Hs01077958_s1 |
| *IFNG* | Interferon, gamma | Hs00989291_m1 |
| *IFNL2* | Interferon, lambda 2 | Hs00820125_g1 |

**Supplementary Table 2: Genes differentially expressed in gt1- versus gt3-infected patients**

| **Entrez Gene ID** | **HGNC Gene Symbol** | **Gene Name** | **Fold Change (Gt1 *vs.* Gt3)** |
| --- | --- | --- | --- |
| 3429 | *IFI27* | Interferon, alpha-inducible protein 27 | 6.0 |
| 6614 | *SIGLEC1* | Sialic acid binding Ig-like lectin 1, sialoadhesin | 4.3 |
| 91543 | *RSAD2* | Radical S-adenosyl methionine domain containing 2 | 3.6 |
| 10964 | *IFI44L* | Interferon-induced protein 44-like | 3.6 |
| 6347 | *CCL2* | Chemokine (C-C motif) ligand 2 | 3.6 |
| 3434 | *IFIT1* | Interferon-induced protein with tetratricopeptide repeats 1 | 3.5 |
| 3437 | *IFIT3* | Interferon-induced protein with tetratricopeptide repeats 3 | 3.5 |
| 3627 | *CXCL10* | Chemokine (C-X-C motif) ligand 10 | 3.4 |
| 3117 | *HLA-DQA1* | Major histocompatibility complex, class II, DQ alpha 1 | 3.3 |
| 710 | *SERPING1* | Serpin peptidase inhibitor, clade G (C1 inhibitor), member 1 | 3.2 |
| 94031 | *HTRA3* | HtrA serine peptidase 3 | 3.1 |
| 83857 | *TMTC1* | Transmembrane and tetratricopeptide repeat containing 1 | 2.8 |
| 1958 | *EGR1* | Early growth response 1 | 2.8 |
| 8638 | *OASL* | 2'-5'-oligoadenylate synthetase-like | 2.7 |
| 7262 | *PHLDA2* | Pleckstrin homology-like domain, family A, member 2 | 2.7 |
| 51513 | *ETV7* | Ets variant 7 | 2.6 |
| 2537 | *IFI6* | Interferon, alpha-inducible protein 6 | 2.5 |
| 3433 | *IFIT2* | Interferon-induced protein with tetratricopeptide repeats 2 | 2.5 |
| 3728 | *JUP* | Junction plakoglobin | 2.4 |
| 10561 | *IFI44* | Interferon-induced protein 44 | 2.4 |
| 9636 | *ISG15* | ISG15 ubiquitin-like modifier | 2.4 |
| 10562 | *OLFM4* | Olfactomedin 4 | 2.4 |
| 9582 | *APOBEC3B* | Apolipoprotein B mRNA editing enzyme, catalytic polypeptide-like 3B | 2.3 |
| 116071 | *BATF2* | Basic leucine zipper transcription factor, ATF-like 2 | 2.3 |
| 200315 | *APOBEC3A* | Apolipoprotein B mRNA editing enzyme, catalytic polypeptide-like 3A | 2.3 |
| 4599 | *MX1* | Myxovirus (influenza virus) resistance 1, interferon-inducible protein p78 (mouse) | 2.2 |
| 2867 | *FFAR2* | Free fatty acid receptor 2 | 2.2 |
| 2353 | *FOS* | FBJ murine osteosarcoma viral oncogene homolog | 2.2 |
| 27074 | *LAMP3* | Lysosomal-associated membrane protein 3 | 2.2 |
| 467 | *ATF3* | Activating transcription factor 3 | 2.2 |
| 4940 | *OAS3* | 2'-5'-oligoadenylate synthetase 3, 100kDa | 2.1 |
| 375790 | *AGRN* | Agrin | 2.1 |
| 820 | *CAMP* | Cathelicidin antimicrobial peptide | 2.1 |
| 759 | *CA1* | Carbonic anhydrase I | 2.1 |
| 4680 | *CEACAM6* | Carcinoembryonic antigen-related cell adhesion molecule 6 (non-specific cross reacting antigen) | 2.1 |
| 118932 | *ANKRD22* | Ankyrin repeat domain 22 | 2.1 |
| 717 | *C2* | Complement component 2 | 2.0 |
| 27087 | *B3GAT1* | Beta-1,3-glucuronyltransferase 1 (glucuronosyltransferase P) | -2.0 |
| 2670 | *GFAP* | Glial fibrillary acidic protein | -2.2 |
| 284013 | *VMO1* | Vitelline membrane outer layer 1 homolog (chicken) | -2.3 |
| 79812 | *MMRN2* | Multimerin 2 | -2.3 |
| 9891 | *NUAK1* | NUAK family, SNF1-like kinase, 1 | -2.4 |
